# Flaccid Lower Limb Paraparesis Despite Infiltration of the Spinal Cord by a Secondary Central Nervous System T-cell Non-Hodgkin Lymphoma With Syringomyelia: A Case Report

**DOI:** 10.7759/cureus.103117

**Published:** 2026-02-06

**Authors:** Josef Finsterer

**Affiliations:** 1 Neurology, Neurology and Neurophysiology Center, Vienna, AUT

**Keywords:** carcinosis, central nervous system affection, non-hodgkin lymphoma, spinal cord, t-cell lymphoma

## Abstract

Secondary central nervous system (CNS) lymphomas typically present with weakness, spasticity, exaggerated tendon reflexes, and pyramidal signs. A patient with progressive flaccid paraparesis of the lower extremities despite carcinomatosis and infiltration of T-cell non-Hodgkin lymphoma (NHL) into the spinal cord has not been described to date.

A 71-year-old man was diagnosed with T-cell NHL not otherwise specified, stage 4B, and an international prognostic index of 5 based on a lymph node biopsy and bone marrow puncture. The patient received three cycles of cyclophosphamide, hydroxydaunorubicin, vincristine, and prednisone (CHOP) chemotherapy, which had to be discontinued due to thrombocytopenia and the onset of paraparesis of the lower extremities. Examination of the paraparesis revealed lymphoma infiltrates in the brain and spinal cord. Despite these CNS lesions, the patient did not exhibit spasticity, pyramidal signs, or increased reflexes; rather, hypotonia and a general absence of reflexes were observed. These were attributed to axonal motor neuropathy due to vincristine toxicity.

T-cell NHL may not be suppressed by CHOP chemotherapy, may spread secondarily to the CNS, and may infiltrate the brain and spinal cord, manifesting as paraparesis, incontinence, muscle hypotonia, and decreased tendon reflexes. Secondary CNS lymphoma does not necessarily have to be accompanied by hyperreflexia, pyramidal signs, and spasticity if the peripheral nerves are severely affected by vincristine toxicity.

## Introduction

Non-Hodgkin lymphoma (NHL) is a malignant disease of the lymphocytes that originates in the lymphatic system and leads to abnormal cell growth and tumor formation in the lymph nodes, spleen, or other organs [1]. The first symptoms typically include painless swelling in the neck, armpits, or groin; fever; severe night sweats; weight loss; fatigue and loss of energy; itching; abdominal pain; and recurrent or persistent infections [1]. NHLs can be classified according to the type of lymphocytes affected (B-cells (85% of cases), T-cells, or natural killer cells) or according to their growth rate as slow-growing (indolent/low-grade) or fast-growing (aggressive/high-grade) tumors [1]. NHL can also be categorized by immunophenotype (CD markers) or by the presence of specific mutations in genes such as MYV or BCL2, or by translocations [2]. The diagnosis is made through histological and immunohistological examinations of lymph nodes, bone marrow, or other tissues, as well as through genetic testing [1,2]. Treatment options include chemotherapy, radiation therapy, or other targeted therapies [3]. The prognosis varies widely but is often favorable when detected early [1].

If treatment is unsuccessful or a patient refuses treatment, NHL can spread to organs that were not affected initially, such as the liver, gastrointestinal tract, skin, lungs, testicles, eyes, salivary glands, or central nervous system (CNS) (secondary CNS lymphoma) [1,4]. Secondary CNS lymphoma is considered a serious complication of NHL, usually associated with aggressive types and often occurring as a relapse or part of a systemic disease [4]. Secondary CNS lymphoma can cause symptoms such as headaches, confusion, personality changes, cognitive changes, memory loss, seizures, dysarthria, visual disturbances (e.g., double vision, blurred vision), motor deficits, cranial nerve palsies, ataxia, spasticity (positive pyramidal signs, increased tendon reflexes), hearing loss, back pain, nausea, vomiting, or problems with urination (e.g., incontinence). Symptoms in the CNS result from involvement of the brain parenchyma, carcinomatosis (spread of the lymphoma to the meninges), increased intracranial pressure, direct effects of the tumor on the brain or spine, or infiltration of the motor roots [4,5]. CNS involvement is diagnosed by clinical neurological examinations, imaging, fluorodeoxyglucose positron emission tomography (FDG-PET), cerebrospinal fluid (CSF) analysis, or brain biopsy [4]. CNS involvement usually manifests as spasticity. A patient with progressive flaccid paraparesis of the lower extremities despite infiltration of T-cell NHL in the brain and spinal cord has not been described to date.

## Case presentation

The patient is a 71-year-old man who was diagnosed with unspecified T-cell NHL, not otherwise specified, stage 4B, with an international prognostic index of 5 based on bone marrow aspiration and lymph node biopsy. Massive bone infiltration was observed. His medical history included dilated cardiomyopathy, tachycardic atrial fibrillation treated with edoxaban and electrocardioversion, heart failure, right bundle branch block, hyperlipidemia, hyperuricemia, and arterial hypertension.

**Figure 1:**
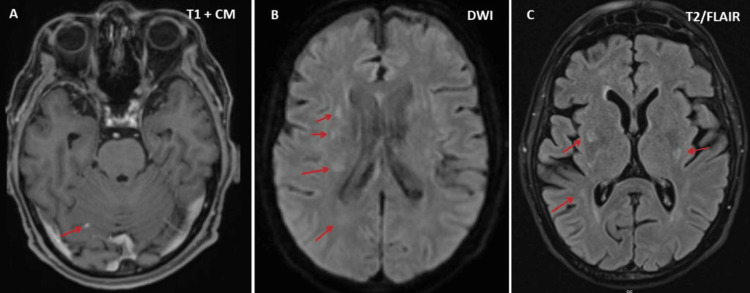
Cerebral MRI showing multiple parenchymal lesions, appearing faintly hyperintense on T2-weighted images (panel C), exhibiting mild diffusion restriction (panel B), and faint contrast enhancement (panel A). The largest supratentorial lesion is located in the periventricular white matter of the right frontal lobe (arrow), with a further lesion in the white matter of the left frontal lobe. Smaller lesions are present subcortically in the left temporal lobe and in the right fronto-basal lobe (arrows)

**Figure 2:**
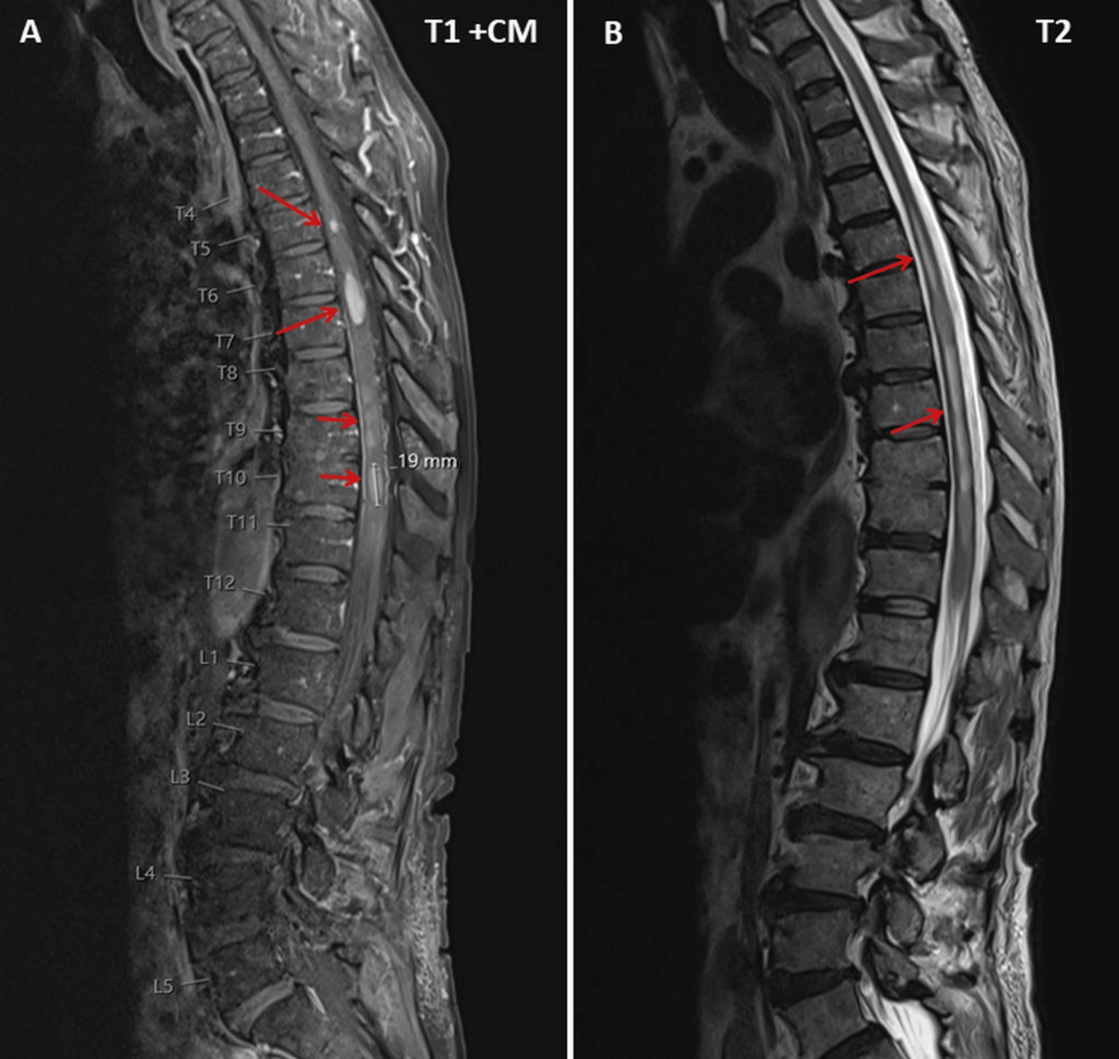
MRI of the thoracic and lumbar spine showing T1 and T2 hyperintense lesions with discrete contrast enhancement in the vertebral bodies of T6, T10, and T11 and multiple oval- to pinhead-shaped, eccentric lesions in the spinal cord at T5 and T11 (panel A, arrows), with accompanying hydromyelia between T4 and T10 (panel B, arrows) MRI: magnetic resonance imaging

**Figure 3:**
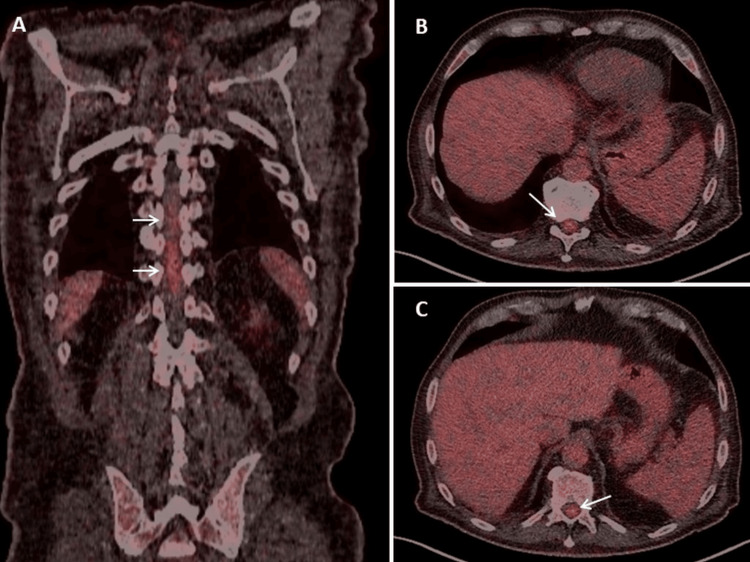
FDG-PET coronary (panel A) and axial planes (panels B and C) showing a linear uptake at the level of T10/T11 in the spinal cord (SUVmax 5; Deauville 5) (arrows), which can be interpreted as either infiltration of the lymphoma or carcinosis FDG-PET: fluorodeoxyglucose positron emission tomography

Two weeks after three cycles of chemotherapy with cyclophosphamide, hydroxydaunorubicin, vincristine (cumulative dosage 6 mg), and prednisone (CHOP), the patient suffered from progressive loss of appetite and slowly progressive lower limb weakness, first in the right and then in the left leg, increasing gait disturbances, and was hospitalized again because he could no longer stand or walk. The chemotherapy was further complicated by a cytomegalovirus (CMV) infection. The clinical neurological examination revealed reduced or absent tendon reflexes in the upper and lower extremities, bladder dysfunction, and flaccidity with Medical Research Council grade 3-4 paraparesis of the lower extremities, without weakness of the upper extremities and without sensory disturbances (preserved superficial and deep sensation). Blood tests revealed anemia, leukopenia, pronounced thrombocytopenia, slightly elevated C-reactive proteins, and hypoproteinemia (Table 1). Magnetic resonance imaging (MRI) of the brain showed multiple lesions suggestive of lymphoma infiltration, both infratentorial and supratentorial (Figure 1). MRI of the thoracic and lumbar spine showed T1- and T2-hyperintense lesions in three vertebral bodies (Figure 2). Additionally, several eccentric lesions were identified between T5 and T11, with hydromyelia extending from T4 to T10 (Figure 2). FDG-PET showed hyperactivity of the spinal cord at the T10-T11 level (Figure 3). Examination of the CSF revealed minimal pleocytosis (6/3; n < 5/3) and elevated protein levels. Histological examination of the cells revealed lymphoma cells, indicating carcinomatosis of the meninges. Nerve conduction studies (NCS) revealed axonal motor neuropathy. His current medication included edoxaban, bisoprolol, digoxin, furosemide/spironolactone, atorvastatin, and allopurinol. The planned fourth cycle of CHOP was postponed due to severe thrombocytopenia (Table 1) and paraparesis. Due to the rapid progression of the disease, it was decided to refer the patient for radiation therapy.

**Table 1:** Results of blood tests during hospitalization ALP: alkaline phosphatase, CRP: C-reactive protein, IgG: immunglobulin G; LDH: lactate dehydrogenase, hd: hospital day, nd: not done, RL: reference limits

Parameter	RL	Hd1	Hd3	Hd6
CRP	0.0-4.9 mg/l	1.6 mg/l	9.6 mg/l	9.2 mg/l
Leukocytes	4-10 G/l	3.6 G/l	6.7 G/l	3.9 G/l
Erythrocytes	4.2-5.5 T/l	3.1 T/l	2.8 T/l	2.8 T/l
Thrombocytes	150-400 G/l	65 G/l	64 G/l	62 G/l
LDH	0-247 U/l	154 U/l	153 U/l	110 U/l
Glucose	70-100 mg/dl	nd	112 mg/dl	82 mg/dl
Total protein	6.6-8.3 g/dl	nd	5.7 g/dl	nd
IgG	700-1500 mg/dl	nd	570 mg/dl	nd
Calcium	2.20-2.65 mmol/l	2.37 mmol/l	2.20 mmol/l	2.27 mmol/l
ALP	30-120 U/l	78 U/l	74 U/l	73 U/l

## Discussion

The patient presented here is interesting in several aspects. First, he was diagnosed with T-cell NHL, a rare subtype of NHL [6]. T-cell NHL is unusual, much rarer than B-cell NHL, and accounts for only 10-15% of all NHL cases in Western countries, with significant geographical differences [7]. T-cell NHL is often associated with viral infections, such as Epstein-Barr virus or human T-lymphotropic virus type 1 (HTLV-1), particularly in Asia [7].

The second interesting point is that CSF examinations revealed only minimal pleocytosis; however, the 6/3 cells were tumor cells, suggesting carcinomatosis. Carcinomatosis can also affect the nerve roots, which may explain the decreased tendon reflexes and flaccidity of the paraparesis [8]. However, one argument against infiltration of the motor roots is that carcinomatosis usually affects not only the motor roots but also the posterior roots. The patient had no sensory deficits, suggesting that the sensory system, including the dorsal roots, was not affected. Another argument against nerve root involvement is the absence of weakness in the upper extremities. Furthermore, no enhancement of the nerve roots was seen on contrast-enhanced MRI of the lumbar spine.

Decreased tendon reflexes and progressive paraparesis could also be explained by selective impairment of motor function due to Guillain-Barré syndrome (GBS) [9]. The trigger for GBS could be the documented CMV infection resulting from immunosuppression due to the CHOP regimen. CMV infection is a trigger for GBS [10]. Arguments in favor of GBS include prior CMV infection, elevated CSF protein levels, and the clinical presentation. However, arguments against GBS include the absence of nerve root enhancement on contrast-enhanced MRI of the spine, the observation that CMV-associated GBS is usually sensorimotor and affects the cranial nerves, and the absence of NCS findings consistent with GBS.

It is also conceivable that the progressive paraparesis of the lower extremities was due to syringomyelia, as this often leads to reduced or absent tendon reflexes due to damage to the anterior horn cells at the level of the syrinx. Another argument in favor of syringomyelia is that it did not extend beyond the T5 level; however, this does not explain the reduced tendon reflexes in the upper extremities. An argument against syringomyelia as the cause of flaccid paraparesis is that sensory disturbances are often associated with syringomyelia and can lead to spasticity when the corticospinal tract is involved. In any case, it cannot be ruled out that the infiltration of the spinal cord by the lymphoma and the syringomyelia contributed to the paraparesis of the lower extremities.

The most likely cause of flaccid paraparesis and generally reduced tendon reflexes, as well as the absence of sensory deficits, is toxic motor neuropathy due to the neurotoxicity of some components of the CHOP regimen, which includes CHOP [11]. Vincristine is known to frequently cause axonal polyneuropathy due to disruption of microtubules, which are essential for axonal integration and transport of materials along the axon [12]. Vincristine can damage motor, sensory, or autonomic fibers in isolation [13]. In a study of 42 pediatric patients with vincristine-associated neuropathy, pure motor involvement was observed in 88% [14]. Cyclophosphamide is also known to cause neuropathies, especially at high doses [15]. Daunorubicin can also cause polyneuropathies, but this is a rare complication that can only occur at high doses [16]. However, one argument against cyclophosphamide- or daunorubicin-induced polyradiculoneuropathy is that these neuropathies are usually sensory-motor, whereas the index patient has exclusive motor involvement [17].

Finally, it is also conceivable that the paraparesis of the lower extremities was due to toxic myopathy caused by the use of glucocorticoids. It is known that steroids can cause myopathy [18]. However, steroid myopathy usually affects both the upper and lower extremities [19]. Additionally, creatine kinase levels were unchanged, and electromyography (EMG) was normal; however, a normal EMG does not generally rule out myopathy [20].

## Conclusions

This case demonstrates that CHOP chemotherapy may be insufficient to suppress T-cell NHL, that T-cell NHL can spread secondarily to the CNS, affecting not only the brain but also the spinal cord, and that spinal cord infiltration can lead to paraparesis and bladder dysfunction. Secondary CNS lymphoma does not necessarily have to be accompanied by hyperreflexia, pyramidal signs, and spasticity. Still, it can also present with flaccid paraparesis with weakened reflexes if the peripheral nerves are additionally severely impaired by toxic axonal neuropathy following chemotherapy. Additionally, neurologists and oncologists should be aware that even minimal pleocytosis can represent carcinomatosis.
